# Sex differences in blood pressure phenotypes over time – the HELIUS study

**DOI:** 10.1097/HJH.0000000000003676

**Published:** 2024-02-12

**Authors:** Esther M.C. Vriend, Thomas A. Bouwmeester, Oscar H. Franco, Henrike Galenkamp, Aeilko H. Zwinderman, Bert-Jan H. van den Born, Didier Collard

**Affiliations:** a Amsterdam UMC, University of Amsterdam, Department of Internal Medicine, Section Vascular Medicine, Amsterdam Cardiovascular Sciences; b Amsterdam UMC, University of Amsterdam, Department of Public and Occupational Health, Amsterdam Public Health Research Institute; c Julius Center for Health Sciences and Primary Care, University Medical Center Utrecht, Utrecht University, Utrecht; d Amsterdam UMC, University of Amsterdam, Department of Epidemiology, Biostatistics & Bioinformatics, Amsterdam, The Netherlands

**Keywords:** blood pressure, blood pressure phenotype, HELIUS study, hypertension, isolated diastolic hypertension, isolated systolic hypertension, systolic diastolic hypertension

## Abstract

**Background:**

Hypertension can be classified into different phenotypes according to systolic and diastolic blood pressure (BP). In younger adults, these phenotypical differences have different prognostic value for men and women. However, little is known about sex differences in the natural course of different BP phenotypes over time.

**Methods:**

We used baseline and follow-up data from the multiethnic, population-based HELIUS study to assess differences in BP phenotypes over time in men and women aged < 45 years stratified according to baseline office BP into normotension (<140/<90 mmHg), isolated systolic hypertension (ISH, ≥140/<90 mmHg), isolated diastolic hypertension (IDH, <140/≥90 mmHg) or systolic diastolic hypertension (SDH, ≥140/≥90 mmHg). Logistic regression adjusted for age, ethnicity, and follow-up time was used to assess the risk of hypertension at follow-up (BP ≥140/90 mmHg or use of antihypertensive medication), stratified by sex.

**Results:**

We included 4103 participants [mean age 33.5 years (SD 7.4), 43.4% men] with a median follow-up time of 6.2 years. Compared to normotensive individuals, the age-adjusted odds ratios (OR) for having hypertension at follow-up were 4.78 (95% CI 2.90; 7.76) for ISH, 6.02 (95% CI 3.70; 9.74) for IDH and 33.73 (95% CI 20.35; 58.38) for SDH in men, while in women, OR were 10.08 (95% CI 4.09; 25.56) for ISH, 27.59 (95% CI 14.68; 53.82) for IDH and 50.58 (95% CI 24.78; 114.84) for SDH.

**Conclusions:**

The risk of hypertension at follow-up was higher among women for all phenotypes compared to men, particularly in those with IDH. Findings of this study emphasize the importance of close BP monitoring in the young, especially in women.

## Graphical abstract

**Figure:**
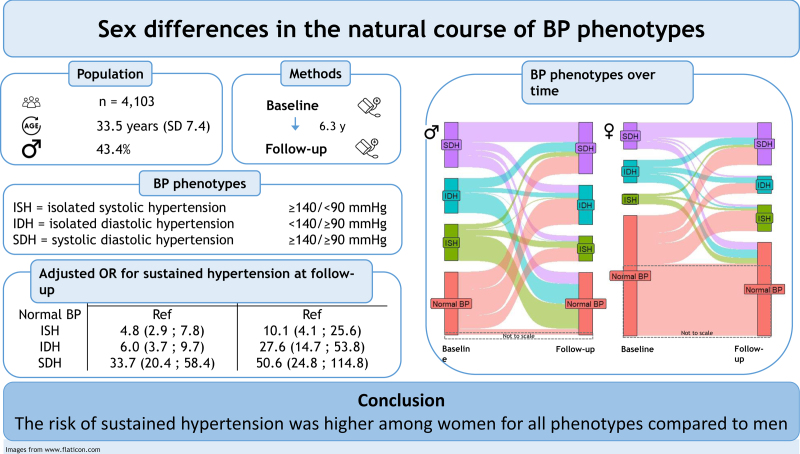

## INTRODUCTION

In clinical practice, hypertension can be subdivided into different phenotypes according to the elevation of systolic (SBP) and diastolic blood pressure (DBP). This differentiation is relevant because isolated systolic hypertension (ISH), isolated diastolic hypertension (IDH) and combined systolic and diastolic hypertension (SDH) carry a different risk of cardiovascular disease (CVD) with important sex differences, especially in younger adults [1–3]. A previous study has shown that young and middle-aged men with ISH have a comparable CVD risk compared to men with high-normal BP, while the risk of CVD in young and middle-aged women with ISH is higher compared to women with high-normal BP [2]. In contrast to the elderly, ISH in young adults is not only associated with increased arterial stiffness, but also with increased stroke volume and higher pulse pressure, which may differentially impact cardiovascular risk [4–7]. There is ongoing debate whether ISH in younger adults is either an innocuous phenomenon or necessitates BP-lowering treatment [1–3,8–10]. In clinical practice, it is important to base treatment and follow-up decisions not only on long-term CVD risk but also on the risk for development of sustained hypertension over time. In middle-aged adults, both IDH and ISH are associated with the development of sustained hypertension later in life, with the strongest association for IDH [11]. However, for younger adults there is a paucity of data on the natural course of different blood pressure phenotypes over time, which complicates the decision of whether close follow-up and treatment is indicated. Moreover, given the considerable variability in blood pressure levels, the prognostic significance of single blood pressure measurements in young individuals remains to be fully elucidated. In this study, we therefore examined sex differences in the risk of hypertension at follow-up for different BP phenotypes in young adult men and women aged <45 years who participated in a large multiethnic population-based study.

## METHODS

### Study design

We used baseline and follow-up data of the HEalthy Life In an Urban Setting (HELIUS) study which has been previously described in detail elsewhere [12]. Briefly, HELIUS is a large-scale multiethnic population-based study designed to research ethnic differences in health and care utilisation in Amsterdam, the Netherlands. Between 2011 and 2015, the HELIUS study recruited 24 781 individuals aged 18 to 70 years from six different ethnic groups (Dutch, Ghanaian, African Surinamese, South-Asian Surinamese Moroccan, and Turkish descent). Using the municipality register, participants were randomly selected, stratified by ethnicity and invited to participate. Follow-up data collection started in 2019 and was finished in 2022. Both the baseline and follow-up study visit consisted of a physical examination, a questionnaire, and collection of biological samples. The HELIUS study is performed in compliance with the Declaration of Helsinki and has been approved by the Ethical Review Board of the Amsterdam UMC, location AMC. All participants provided written informed consent.

### Definitions and measurements

Demographics, history of CVD (defined as self-reported stroke, myocardial infarction and coronary or peripheral revascularization) and information on smoking was obtained using questionnaires. Ethnicity was determined based on the country of birth of the participant and that of their parents. Questionnaires were used to distinguish between African Surinamese, South-Asian Surinamese or other Surinamese participants. Blood samples were collected to determine the concentration of glucose, HbA1C, creatinine, and total cholesterol after 10–14 h of fasting. Diabetes mellitus was defined as either elevated fasting glucose levels (≥7 mmol/l) and/or use of glucose lowering medication. Body mass index was calculated by weight (in kg) divided by height (in m^2^). Estimated glomerular filtration rate (eGFR) was calculated with the revised CKD-EPI 2021 creatinine equation [13]. BP was measured in the same way at baseline and at follow-up by measuring BP twice. SBP and DBP at each visit were determined as the mean of the two measurements. Measurements were taken seated on the left arm after at least five minutes rest using a validated semi-automatic oscillometric device (Microlife WatchBP Home; Microlife AG, Switzerland). Participants were asked to bring all prescribed medication to the research location. The use of antihypertensive medication was determined based on the Anatomic Therapeutic Chemical classification, including beta-blockers.

### Statistical analysis

Participants were stratified according to their BP phenotype at baseline, subdividing them into individuals with normotension (SBP<140 mmHg, DBP<90 mmHg), ISH (SBP≥140 mmHg, DBP<90 mmHg), IDH (SBP<140 mmHg, DBP≥90 mmHg) and SDH (SBP≥140 mmHg, DBP≥90 mmHg) according to current European guidelines [14]. We used data of participants aged <45 years at baseline. This cut off value was chosen to align the mean age with that of previous publications [2]. Participants using BP lowering medication at baseline were excluded. Demographic and clinical characteristics were expressed as percentage, mean (SD) or median [IQR] and compared between the hypertensive subtypes using the analysis of variance, Kruskal–Wallis and chi-square tests, stratified by sex. The change in BP phenotypes over time were visualised using a Sankey diagram. A scatterplot was created to illustrate the baseline SBP and DBP levels for each participant, with each participant classified as either normotensive or hypertensive at follow-up.

We calculated the age-adjusted absolute risk of hypertension at follow-up (defined as SBP≥140 mmHg, DBP≥90 mmHg and/or use of antihypertensive medication at follow-up) for each blood pressure phenotype in both men and women. To examine the association between hypertensive subtype at baseline and the risk of hypertension at follow-up, we calculated odds ratios and 95% confidence intervals using logistic regression models adjusted for age, sex and follow-up time with normotensive individuals as reference. To explore differences between men and women in the progression of BP phenotypes over time, we compared a model with and without an interaction term between sex and BP phenotype at baseline. In model 2, we further adjusted for possible confounders by adding baseline BMI, time-dependent change in BMI, baseline eGFR, diabetes mellitus, and total cholesterol [15]. Change in SBP was calculated for each phenotype using linear regression models with normotensive individuals as reference, corrected for baseline SBP and with addition of the same variables as in the logistic regression models. Further adjustment for baseline DBP, did not materially change these results (data not shown). In the linear regression analysis for SBP change over time, SBP levels at follow-up were corrected for the use of antihypertensive medication by adding 10 mmHg following previous literature [16,17]. All regression models were stratified by sex.

Sensitivity analyses were performed to compare the complete cases analysis with results obtained by applying different methods to correct for loss to follow-up (imputation and inverse probability weighted regression analyses). Imputation was performed in participants without follow-up data (*n* = 5850) using multivariate imputation by chained equations (MICE) with the following variables at baseline: age, BP levels, antihypertensive medication, history of CVD, BMI, eGFR, smoking, and follow-up time. Inverse probability weighting was performed using a logistic regression model that included baseline SBP, DBP, age, ethnicity, smoking status, presence of diabetes mellitus, total cholesterol levels, and history of CVD. We considered two-sided probability values of 0.05 as statistically significant. Data analysis was performed in R version 4.0.1 (Vienna, Austria) using the nlme (version 3.1–159), tableone (version 0.13.2) and MICE packages (version 3.14.0).

## RESULTS

### Selection of participants

**FIGURE 1:**
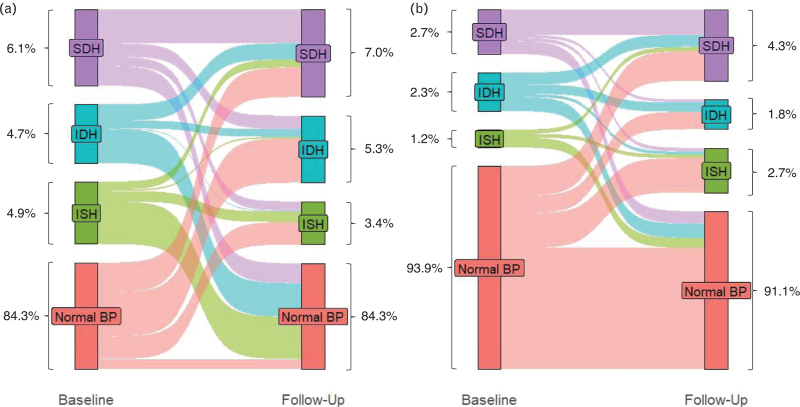
Sankey diagram of changes in hypertensive phenotypes over time for men (a) and women (b). Bars corresponding to normotensive participants at both baseline and follow-up were partly omitted as indicated by the discontinuous y-axis. The percentages represent the distribution of blood pressure phenotypes at baseline and follow-up, respectively. IDH, isolated diastolic hypertension; ISH, isolated systolic hypertension; SDH, systolic diastolic hypertension.

Of the 24 781 HELIUS participants, 10 293 participants aged <45 years at baseline were selected without missing data on the questionnaires and physical examination (Figure 1, Supplemental Digital Content). After exclusion of participants with baseline use of BP lowering medication (*n* = 340), 9953 participants with complete baseline data were eligible for inclusion. Of these participants, 4103 attended the follow-up visit (response rate 41.0%) with a median follow-up time of 6.2 years (SD 1.1) and were included in the complete case analysis.

### Study population

Baseline characteristics of the included population stratified by BP phenotype are presented in Table 1. In total, 1781 men and 2322 women were included with a median age of 32.0 years [interquartile range (IQR) 26.0–39.0]. All hypertensive phenotypes were more prevalent in men then in women. Compared to normotensives at baseline, all hypertensive phenotypes exhibited a higher mean BMI, higher mean total cholesterol, and a higher proportion of individuals with diabetes mellitus or a history of cardiovascular disease (CVD). The overall prevalence of a positive history of CVD at baseline was low (*n* = 38, 1.0% of the total population) and was slightly increased at follow-up (*n* = 71, 1.7%). At follow-up, SBP levels (uncorrected for use of BP lowering medication) decreased in all phenotypes for both men and women, except for men and women with IDH. Here, SBP increased from 132.6 to 133.6 mmHg in men and 133.4 to 136.0 mmHg in women. At follow-up, 3.5% of all participants used BP-lowering medication (3.4% in men, 3.6% in women), with the lowest amount of participants using diuretics (0.1%) and the highest amount of participants using agents acting on the renin-angiotensin system (1.6%). Baseline characteristics of the population stratified by the availability of follow-up data can be found in Table 1, Supplemental Digital Content. In both men and women, participants with available follow-up data were older, more frequently of Dutch or South-Asian origin and had slightly higher BP levels.

**TABLE 1:** Characteristics of the included population at baseline, stratified by sex and hypertensive phenotype

		Men				Women			
		NT	ISH	IDH	SDH	NT	ISH	IDH	SDH
		*n* = 1502	*n* = 88	*n* = 83	*n* = 108	*n* = 2180	*n =* *25*	*n =* *54*	*n =* *63*
Age (years), median [IQR]		34 [28–39]	32 [26–39]	38 [33–42]	39 [35–42]	34 [27–40]	40 [36–42]	37 [32–41]	40 [36–42]
Ethnicity, n (%)	Dutch	454 (30.2)	27 (30.7)	20 (24.1)	27 (25.0)	566 (26.0)	2 (8.0)	8 (14.8)	2 (3.2)
	SA Surinamese	248 (16.5)	11 (12.5)	12 (14.5)	18 (16.7)	307 (14.1)	1 (4.0)	10 (18.5)	10 (15.9)
	A Surinamese	166 (11.1)	11 (12.5)	20 (24.1)	17 (15.7)	328 (15.0)	3 (12.0)	16 (29.6)	23 (36.5)
	Ghanaian	69 (4.6)	8 (9.1)	2 (2.4)	15 (13.9)	154 (7.1)	13 (52.0)	11 (20.4)	21 (33.3)
	Turkish	220 (14.6)	11 (12.5)	18 (21.7)	15 (13.9)	299 (13.7)	2 (8.0)	3 (5.6)	2 (3.2)
	Moroccan	315 (21.0)	19 (21.6)	9 (10.8)	15 (13.9)	480 (22.0)	4 (16.0)	6 (11.1)	5 (7.9)
	Other	30 (2.0)	1 (1.1)	2 (2.4)	1 (0.9)	46 (2.1)	0 (0.0)	0 (0.0)	0 (0.0)
SBP (mmHg), mean (SD)		121.7 (8.5)	145.1 (4.6)	132.6 (4.3)	153.4 (11.6)	113.3 (9.9)	145.1 (4.6)	131.4 (5.6)	154.7 (16.1)
DBP (mmHg), mean (SD)		76.4 (6.5)	85.1 (5.1)	92.4 (2.4)	98.6 (7.1)	71.3 (7.1)	85.1 (5.1)	92.4 (2.8)	99.2 (7.0)
BMI (kg/m^2^), mean (SD)		24.9 (3.6)	27.2 (3.8)	26.6 (3.6)	28.8 (4.5)	25.2 (4.8)	30.6 (5.3)	28.6 (5.9)	31.0 (6.2)
Diabetes mellitus^a^, n (%)		18 (1.2)	0 (0.0)	5 (6.0)	6 (5.6)	17 (0.8)	0 (0.0)	3 (5.6)	3 (4.8)
Total chol. (mmol/l), mean (SD)		4.7 (1.0)	4.7 (0.8)	5.2 (0.9)	5.3 (0.9)	4.6 (0.8)	4.7 (0.8)	4.9 (0.8)	4.6 (0.8)
Smoking (yes), n (%)		420 (28.1)	27 (30.7)	28 (34.1)	24 (22.4)	426 (19.6)	2 (8.0)	4 (7.4)	8 (12.7)
History of CVD^b^, n (%)		18 (1.2)	0 (0.0)	2 (2.4)	3 (2.8)	12 (0.6)	0 (0.0)	1 (1.9)	2 (3.2)

A, African; BMI, body mass index; Chol., cholesterol; CVD, cardiovascular diseases; DBP, diastolic blood pressure; IQR, interquartile range; SA, South-Asian; SBP, systolic blood pressure; SD, standard deviation.

a Diabetes mellitus is based on increased fasting glucose levels (≥7 mmol/l) and/or use of glucose lowering medication.

b History of CVD is based on self-reported stroke, myocardial infarction and coronal or peripheral revascularization.

### Change in BP phenotypes over time

**FIGURE 2:**
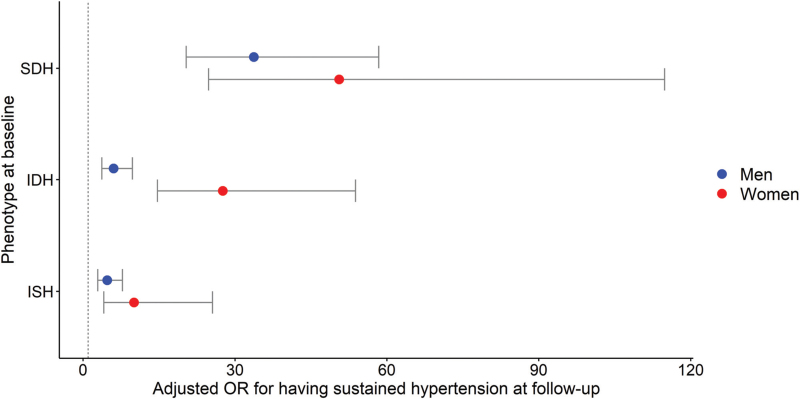
Risk of having hypertension at follow-up (SBP ≥ 140 mmHg, DBP ≥ 90 mmHg and/or use of antihypertensive medication) at follow-up compared to normotensives for the different hypertensive subtypes at baseline. IDH, isolated diastolic hypertension; ISH, isolated systolic hypertension; OR, odds ratio; SDH, systolic diastolic hypertension.

The proportional change between baseline and follow-up according to the different BP phenotypes stratified by sex is shown in Fig. 1, absolute numbers can be found in Table 2. In men, the majority of participants with ISH and IDH at baseline were normotensive at follow-up, whereas in women the majority of the participants with IDH were hypertensive at follow-up. In both men and women, a significant portion of individuals with SDH at baseline remained hypertensive at follow-up. Baseline systolic and diastolic BP levels according to BP phenotype are shown in Figure 2, Supplemental Digital Content.

**TABLE 2:** Change in hypertensive phenotypes over time for men (A) and women (B)

A						
		Phenotype at follow-up				
		Normal BP	ISH	IDH	SDH	Total
Phenotype at baseline	Normal BP	1365 (90.9)	32 (2.1)	63 (4.2)	42 (2.8)	1502 (100.0)
	ISH	60 (68.2)	15 (17.0)	2 (2.3)	11 (12.5)	88 (100.0)
	IDH	48 (57.8)	1 (1.2)	11 (13.3)	23 (27.7)	83 (100.0)
	SDH	28 (25.9)	13 (12.0)	19 (17.6)	48 (44.4)	108 (100.0)
	Total	1501	61	95	124	1781

B						
		Phenotype at follow-up				
		Normal BP	ISH	IDH	SDH	Total
Phenotype at baseline	Normal BP	2064 (94.7)	49 (2.2)	25 (1.1)	42 (1.9)	2180 (100.0)
	ISH	14 (56.0)	5 (20.0)	0 (0.0)	6 (24.0)	25 (100.0)
	IDH	20 (37.0)	4 (7.4)	13 (24.1)	17 (31.5)	54 (100.0)
	SDH	18 (28.6)	5 (7.9)	4 (6.3)	36 (57.1)	63 (100.0)
	Total	2116	63	42	101	2322

BP, blood pressure; IDH, isolated diastolic hypertension; ISH, isolated systolic hypertension; SDH, systolic diastolic hypertension.

### Risk of hypertension at follow-up and change in SBP according to blood pressure phenotype

Age-adjusted risk for the development of hypertension is depicted in Table 3. The risk of hypertension at follow-up was highest in women for all hypertensive phenotypes compared to men. There was a significant interaction between sex and BP phenotype in the risk of having hypertension at follow-up (*P* < 0.001). The adjusted odds-ratio (OR) for having hypertension at follow-up are depicted in Fig. 2 and Table 2, Supplemental Digital Content. In both sexes, the adjusted OR having hypertension at follow-up were the lowest for ISH and the highest for SDH when compared to normotensive individuals. In women, the OR for IDH was higher (27.59, 95% CI 14.68; 53.82) compared to men (6.02, 95% CI 3.70; 9.74). Further adjustment for other CVD risk factors, did not significantly alter the differences in the risk of hypertension at follow-up. Baseline BMI and change in BMI were the CVD risk factors most strongly associated with the risk of hypertension at follow-up. Table 3, Supplemental Digital Content shows the results of the linear regression analysis examining the change in SBP levels over time according to blood pressure phenotype. In both men and women, no significant difference in change in SBP was observed between participants with ISH and those who were normotensive at baseline. The increase in SBP was similar among men and women with SDH, while women with IDH exhibited the highest increase in SBP compared to normotensives (9.70 mmHg, 95% CI 6.63; 12.78). Sensitivity analysis on different methods to correct for loss to follow-up are shown in Table 4, Supplemental Digital Content. Complete case analysis and inverse probability weighting regression analysis were performed using data of 4103 participants, whereas imputation was conducted using data of 9953 participants. Weighted regression analysis and imputation yielded similar results compared to the complete case analysis, with the narrowest confidence intervals in the weighted regression model.

**TABLE 3:** Age-adjusted absolute risk and its 95% confidence interval of having hypertension (SBP ≥ 140 mmHg, DBP ≥ 90 mmHg and/or use of antihypertensive medication) at follow-up for the different hypertensive subtypes at baseline

	Men *n* = 1781	Women *n* = 2322
Normotensives	10.2 (8.6; 11.9)	6.4 (5.3; 7.5)
ISH	34.1 (22.6; 50.1)	40.8 (20.8; 100.0)
IDH	42.6 (27.9; 65.4)	62.6 (42.2; 96.7)
SDH	74.5 (57.2; 97.8)	85.7 (55.1; 100.0)

IDH, isolated diastolic hypertension; ISH, isolated systolic hypertension; SDH, systolic diastolic hypertension.

## DISCUSSION

We found important sex differences in the risk of hypertension at follow-up according to BP phenotype in younger adults. Although all hypertensive phenotypes were associated with an increased risk of hypertension, the risk was considerably higher in women compared to men, with the most pronounced sex differences in those with IDH. In both men and women, individuals with ISH had a slightly increased risk of having hypertension at follow-up when compared to normotensives. Individuals with SDH had the highest risk of having hypertension at follow-up in both men and women. Adjustment for confounding factors did not materially influence the association with different hypertensive phenotypes.

Consistent with literature, we found higher prevalence rates of all hypertensive subtypes in men [4,5,7,10,18]. Sex-specific analyses of BP among 32 833 participants from four different population-based cohort studies have shown that men have higher BP levels early in life whereas BP increases more rapidly in women later on, bridging the difference between men and women by the 6th decade in life [15]. It is likely that the progressive BP rise with age in women explains the higher odds of hypertension at follow-up according to different BP phenotype. Hormonal and morphological differences in the cardiovascular system between men and women could play a role in the underlying causes of these sex differences [19]. Alternatively, it can be argued that the presence of ISH or IDH in women is more specific for developing hypertension at follow-up because normal BP values in young women are generally lower than in young men, and – as a consequence – less susceptible to regression to the mean.

There is a paucity of data on the natural course of hypertensive phenotypes over time in younger adults, especially in relation to differences between men and women. The Framingham Heart study, for example, examined the risk of new-onset SDH for different hypertensive phenotypes in 3915 participants included between 1953 and 1967 [11]. Compared to optimal BP, those with IDH had the highest HR for new-onset SDH over a 10-year follow-up period with an adjusted hazard ratio (HR) of 23.2, whereas for those with ISH adjusted HR to develop hypertension at follow-up was 7.10. However, the higher mean age of the population (45 years) and the increasing prevalence of hypertension among young adults during the past five decades may affect generalizability of the results to a younger and current day population [20]. A more recent study, including 1140 participants (aged 18–45 years) from the HARVEST study with a comparable median follow-up time to our study, performed analyses using both office and ambulatory BP levels. In this study, individuals with ISH had the lowest risk of having hypertension at follow-up requiring BP-lowering treatment, followed by individuals IDH and SDH, which aligns with the results of our study [21]. However, it is important to note that both studies did not perform stratified analyses based on sex.

The prognostic significance of hypertensive phenotypes in young adults, especially ISH, remains a subject of debate [8,9]. Various researchers have postulated that ISH among the young is a benign condition caused by increased pulse pressure amplification [4,5], while others have claimed that, also in young adults, ISH is caused by stiffening of large arteries [7,22]. The substantial proportion of participants who transitioned from ISH to normotension during follow-up and the lack of a progressive SBP increase over time in individuals with ISH supports the hypothesis that ISH carries an intermediate risk, as they still showed a higher risk of having hypertension at follow-up compared to those who were initially normotensive. To discriminate between ISH with and without an increased risk of CVD, it has been suggested to measure central BP in these subjects, since central BP is known to be superior to brachial pressure in correlating with adverse outcomes [23,24]. Indeed, previous studies have found that ISH with low central BP levels is associated with a lower risk of future hypertension compared to individuals with ISH and high central BP [25]. However, since there is no gold standard to measure central BP noninvasively, the applicability of central BP in a clinical and research setting remains limited.

In line with other studies, our study shows that individuals with IDH have a high risk to develop hypertension as is also shown by the large increase in SBP in both men and women with IDH at baseline. Increased peripheral vascular resistance is considered as the main pathophysiological mechanism underlying IDH and it is argued that the process underlying the frequent progression towards SDH results from progressive large artery stiffening [26]. In our study, both men and women with IDH had a higher BMI and higher prevalence of smoking compared to individuals with normal BP. Furthermore, we found that participants with IDH, regardless of sex, exhibited the most pronounced increase in BMI over time when compared to individuals with other phenotypes. Prior research has shown that obesity and metabolic syndrome increase the risk of all hypertensive phenotypes, especially in individuals with IDH [27,28]. This is also in line with previous studies that have reported a higher CVD risk in individuals with IDH [11,29] and further supported by the increased CVD risk in men and women with IDH in the Chicago Heart Association study [2]. Here, IDH in both men and women aged 18–49 years at baseline was associated with increased CVD mortality after an average of 31 years follow-up. Data from a National Health Insurance Database in Korea, demonstrated that individuals with ISH and IDH have an equally elevated risk of CVD after a follow-up period of 13.2 years [3]. The average age of the participants in their study was similar to that of our study (30 years), however, their analyses were not stratified by sex. Another cohort study, which included 1,207,141 young men, found a stronger association between DBP and CVD compared to SBP [30]. However, the question remains whether the increased risk of CVD are caused by ISH or DBP per se, or if they are a result of the increased likelihood of having hypertension over time that is associated with IDH as demonstrated in our study.

Strengths of this study include the use of a large-scale, multiethnic population-based cohort study, allowing us to examine the natural course of BP phenotypes over time in a young, diverse population. BP measurements were consistently obtained using a standardized approach at both baseline and follow-up. Limitations of this study include the loss to follow-up. Combined with the low prevalence of hypertension among youths and the relatively small group of participants with both baseline and follow-up data, the amount of participants with hypertension was relatively small, limiting the power of the analysis. However, the results were not materially different when data were imputed or inverse probability-weighted regression analysis was used. Another limitation includes the classification of hypertension which was based on office BP measurements at the baseline and follow-up visit. Although we determined BP as the average of two consecutive measurements, the use of office BP measurements can lead to misclassification, including the white coat effect which increases SBP by a greater extent than DBP. However, the white coat effect could not explain the observed sex differences in the prevalence of hypertension at follow-up, since previous research has shown that white coat hypertension is more prevalent in women [31]. To mitigate the white coat effect, ambulatory BP measurements are preferred, but its application on a broad scale is logistically challenging. Besides, visit-to-visit variability and regression to the mean may result in both over and underestimation of BP and will affect SBP more than DBP because of its increased variability [28,32]. Finally, the absence of data regarding the compliance of participants in use of BP lowering medication introduces a potential source of bias that might have influenced the results.

Since current CVD risk estimations are based on 10-year atherosclerotic disease risk calculators, almost all hypertensive young individuals are classified as low risk [14,33,34]. We show important sex differences in the progression to hypertension at follow-up with a much higher risk in women as compared to men, particularly in those with IDH. Although younger women have a low cardiovascular risk, the present findings show that young women with high BP should remain closely monitored during follow-up in clinical practice, regardless of hypertensive phenotype. The strong association between an increase in BMI and the progression to hypertension at follow-up further supports the use of lifestyle measures such as weight reduction to prevent development of hypertension. Further research is required to assess the benefits of antihypertensive treatment in young hypertensive adults.

## ACKNOWLEDGEMENTS

The HELIUS study is conducted by the Academic Medical Center Amsterdam and the Public Health Service of Amsterdam. The HELIUS study is also funded by the Dutch Heart Foundation, the Netherlands Organisation for Health Research and Development, the European Union (FP-7), and the European Fund for the Integration of non-EU immigrants (EIF).

Declaration of conflicting interests: The author(s) declared no potential conflicts of interest with respect to the research, authorship, and/or publication of this article.

Data sharing statement: The HELIUS data are owned by the Amsterdam University Medical Center, location AMC in Amsterdam, The Netherlands. Any researcher can request the data by submitting a proposal to the HELIUS Executive Board as outlined at http://www.heliusstudy.nl/en/researchers/collaboration.

Author contribution: E.M.C.V., D.C., and B.vd.B. contributed to the conception or design of the work. E.M.C.V., T.A.B., A.H.Z., B.vd.B., and D.C. contributed to the acquisition, analysis, or interpretation of data for the work. E.M.C.V. drafted the manuscript. E.M.C.V., T.A.B., O.F., H.G., A.H.Z., B.vd.B., and D.C. critically revised the manuscript. All gave final approval and agree to be accountable for all aspects of work ensuring integrity and accuracy.

### Conflicts of interest

There are no conflicts of interest.

## Supplementary Material

Supplemental Digital Content
