# Supplementary material for: Sex differences in blood pressure phenotypes over time – the HELIUS study
Source: J Hypertens. 2024 Feb 12;42(6):977–83. doi: 10.1097/HJH.0000000000003676 (PMC11064915; doi:10.1097/HJH.0000000000003676)
Supplement: Supplemental Digital Content [file jhype-42-0977-s001.docx]

|  | | **Men** | | **Women** | |
| --- | --- | --- | --- | --- | --- |
|  | | With follow-up | Without follow-up | With follow-up | Without follow-up |
|  | | *n = 1,781* | *n = 2,298* | *n = 2,322* | *n = 3,552* |
| Age (years), median [IQR] | | 35.0 [28.0 – 40.0] | 31.0 [25.0 – 38.0] | 35.0 [27.0 – 40.0] | 31.0 [24.0 – 38.0] |
| Ethnicity,  n (%) | Dutch | 528 (29.6) | 353 (15.4) | 578 (24.9) | 507 (14.3) |
|  | SA Surinamese | 289 (16.2) | 299 (13.0) | 328 (14.1) | 309 ( 8.7) |
|  | A Surinamese | 214 (12.0) | 322 (14.0) | 370 (15.9) | 432 (12.2) |
|  | Ghanaian | 94 ( 5.3) | 186 ( 8.1) | 199 ( 8.6) | 410 (11.5) |
|  | Turkish | 264 (14.8) | 626 (27.2) | 306 (13.2) | 859 (24.2) |
|  | Moroccan | 358 (20.1) | 472 (20.5) | 495 (21.3) | 979 (27.6) |
|  | Other | 34 ( 1.9) | 40 ( 1.7) | 46 ( 2.0) | 56 ( 1.6) |
| SBP (mmHg), mean (SD) | | 125.2 (12.3) | 125.0 (12.1) | 115.2 (12.8) | 115.0 (12.8) |
| DBP (mmHg), mean (SD) | | 78.8 (8.9) | 78.6 (9.3) | 72.7 (9.0) | 72.3 (9.1) |
| BMI (kg/m^2^), mean (SD) | | 25.3 (3.8) | 25.8 (4.4) | 25.5 (5.1) | 26.0 (5.7) |
| Diabetes mellitus^a^, n (%) | | 33 (1.9) | 56 ( 2.5) | 23 ( 1.0) | 44 (1.2) |
| Total chol. (mmol/l), mean (SD) | | 4.8 (1.0) | 4.7 (1.0) | 4.6 (0.8) | 4.5 (0.9) |
| History of CVD^b^ , n (%) | | 24 (1.4) | 47 (2.1) | 15 (0.7) | 45 ( 1.3) |

**Supplementary table 1: Characteristics of the included population at baseline, stratified by sex and the availability of follow-up data.**

*SA = South-Asian. A = African. SBP = systolic blood pressure. DBP = diastolic blood pressure. BMI = body mass index. Chol. = cholesterol. CVD = cardiovascular diseases.* *IQR = interquartile range. SD = standard deviation.*

^a^ Diabetes mellitus is based on increased fasting glucose levels (≥7 mmol/l) and/or use of glucose lowering medication.

^b^ History of CVD is based on self-reported stroke, myocardial infarction and coronal or peripheral revascularization.

|  | **Men** | | **Women** | |
| --- | --- | --- | --- | --- |
|  | Model 1  OR  OOR | Model 2 | Model 1 | Model 2 |
| **Phenotype at baseline** | OR (95% CI) | OR (95% CI) | OR (95% CI) | OR (95% CI) |
| ISH | 4.78 (2.90 ; 7.76)* | 4.60 (2.74 ; 7.63)* | 10.08 (4.09 ; 25.56)* | 7.02 (2.28 ; 17.98)* |
| IDH | 6.02 (3.70 ; 9.74)* | 6.24 (3.75 ; 10.35)* | 27.59 (14.68 ; 53.82) * | 25.82 (13.44 ; 51.54)* |
| SDH | 33.73 (20.35 ; 58.38)* | 32.65 (19.01 ; 58.56) * | 50.58 (24.78 ; 114.84)* | 41.84 (20.27 ; 95.90)* |
| **Age (y)** | 1.06 (1.04 ; 1.08)* | 1.07 (1.04 ; 1.09)* | 1.09 (1.06 ; 1.12)* | 1.08 (1.05 ; 1.11)* |
| **Ethnicity** |  |  |  |  |
| South-Asian Surinamese | 1.09 (0.69 ; 1.71) | 0.79 (0.49 ; 1.28) | 3.03 (1.68 ; 5.58)* | 2.02 (1.07 ; 3.88)* |
| African Surinamese | 0.94 (0.58 ; 1.53) | 0.72 (0.42 ; 1.19) | 3.62 (2.08 ; 6.48) * | 2.23 (1.20 ; 4.23)* |
| Ghanaian | 1.94 (1.06 ; 3.49)* | 1.59 (0.84 ; 2.95) | 5.60 (3.08 ; 10.37)* | 3.37 (1.70 ; 6.80)* |
| Turkish | 1.08 (0.68 ; 1.71) | 0.65 (0.38 ; 1.09) | 2.21 (1.14 ; 4.28) | 1.34 (0.65 ; 2.76) |
| Moroccan | 0.87 (0.56 ; 1.34) | 0.63 (0.39 ; 1.01) | 1.35 (0.71 ; 2.58) | 0.82 (0.40 ; 1.69) |
| Other | 2.01 (0.76 ; 4.76) | 1.69 (0.62 ; 4.16) | 1.46 (0.26 ; 5.34) | 1.01 (0.15 ; 3.86) |
| **BMI (kg/m^2^)** |  | 1.10 (1.05 ; 1.14)* |  | 1.11 (1.08 ; 1.15)* |
| **Change in BMI (kg/m^2^)** |  | 1.28 (1.18 ; 1.38)* |  | 1.10 (1.03 ; 1.18)* |
| **eGFR (ml/min/1.73 m^2^)** |  | 0.99 (0.98 ; 1.01) |  | 1.00 (0.99 ; 1.01) |
| **Smoking** |  |  |  |  |
| Former |  | 0.96 (0.64 ; 1.43) |  | 0.94 (0.52 ; 1.63) |
| Yes |  | 1.06 (0.74 ; 1.51) |  | 1.15 (0.71 ; 1.83) |
| **Diabetes mellitus^a^** |  | 1.36 (0.50 ; 3.45) |  | 0.51 (0.12 ; 1.90) |
| **Total cholesterol (mmol/l)** |  | 0.92 (0.78 ; 1.09) |  | 1.06 (0.86 ; 1.30) |

**Supplementary table 2: Logistic regression analysis on the risk of having hypertension at follow-up (SBP ≥ 140 mmHg, DBP ≥ 90 mmHg and/or use of antihypertensive medication) for the different hypertensive subtypes at baseline compared to normotensives, stratified by sex.** Model 1: adjusted for age, sex, ethnicity and follow-up time. Model 2: model 1 + BMI, eGFR, smoking, diabetes mellitus and total cholesterol levels (all at baseline). *ISH = isolated systolic hypertension. IDH = isolated diastolic hypertension. SDH = systolic diastolic hypertension. SA = South Asian Surinamese. A = African. BMI = body mass index. eGFR = estimated glomerular filtration rate. OR = odds ratio. CI = confidence interval. * Statistically significant (P < 0.05). ^a^Diabetes mellitus is defined as elevated fasting glucose levels (≥7 mmol/l) and/or use of glucose lowering medication.*

|  | **Men** | | **Women** | |
| --- | --- | --- | --- | --- |
|  | Model 1 | Model 2 | Model 1 | Model 2 |
| **Phenotype at baseline** | β (95% CI) | β (95% CI) | β (95% CI) | β (95% CI) |
| ISH | -0.16 (-2.73 ; 2.41) | 0.28 (-2.21 ; 2.77) | 3.43 (-1.16 ; 8.00) | 3.17 (-1.26 ; 7.59) |
| IDH | 4.80 (2.40 ; 7.20)* | 4.99 (2.65 ; 7.33)* | 9.70 (6.63 ; 12.78)* | 9.63 (6.65 ; 12.60)* |
| SDH | 5.32 (2.58 ; 8.06)* | 5.46 (2.80 ; 8.12)* | 5.05 (1.72 ; 8.37)* | 6.20 (2.97 ; 9.43)* |
| **Age (y)** | 0.15 (0.09 ; 0.22)* | 0.19 (0.11 ; 0.27)* | 0.32 (0.27 ; 0.37)* | 0.27 (0.21 ; 0.34)* |
| **Ethnicity** |  |  |  |  |
| South-Asian Surinamese | 0.25 (-1.24 ; 1.74) | -1.25 (-2.76 ; 0.25) | 1.51 (0.02 ; 3.00)* | -0.72 (-2.24 ; 0.81) |
| African Surinamese | 1.97 (0.32 ; 3.63)* | 0.96 (-0.68 ; 2.59) | 4.16 (2.71 ; 5.61)* | 1.78 (0.22 ; 3.35)* |
| Ghanaian | 5.08 (2.79 ; 7.37)* | 4.09 (1.81 ; 6.38)* | 6.36 (4.52 ; 8.20)* | 3.45 (1.47 ; 5.42)* |
| Turkish | 1.02 (-0.52 ; 2.55) | -0.51 (-2.09 ; 1.08) | 0.85 (-0.67 ; 2.37) | -1.04 (-2.63 ; 0.56) |
| Moroccan | 1.49 (0.09 ; 2.88)* | 0.39 (-1.04 ; 1.83) | 0.88 (-0.44 ; 2.19) | -1.61 (-3.12 ; -0.10) |
| Other | 1.48 (-2.11 ; 5.08) | 0.78 (-2.70 ; 4.26) | 2.61 (-0.69 ; 5.91) | 0.89 (-2.33 ; 4.10) |
| **Baseline SBP (mmHg)** | -0.38 (-0.42 ; -0.31)* | -0.39 (-0.44 ; -0.33)* | -0.29 (-0.33 ; -0.24)* | -0.35 (-0.39 ; -0.30)* |
| **BMI (kg/m^2^)** |  | 0.25 (0.11 ; 0.39)* |  | 0.42 (0.31 ; 0.52)* |
| **Change in BMI (kg/m^2^)** |  | 1.40 (1.16 ; 1.65)* |  | 1.05 (0.87 ; 1.23)* |
| **eGFR (ml/min/1.73 m^2^)** |  | -0.03 (-0.07 ; 0.01) |  | -0.02 (-0.06 ; 0.02) |
| **Smoking** |  |  |  |  |
| Former |  | -0.60 (-1.87 ; 0.66) |  | -1.70 (-3.08 ; -0.32) |
| Yes |  | 0.03 (-1.10 ; 1.16) |  | -0.73 (-1.91 ; 0.44) |
| **Diabetes mellitus^a^** |  | 2.25 (-1.29 ; 5.80) |  | -0.07 (-4.47 ; 4.34) |
| **Total cholesterol (mmol/l)** |  | -0.34 (-0.87 ; 0.18) |  | 0.48 (-0.07 ; 1.0) |

**Supplementary table 3: Linear regression analysis on the change in SBP levels over time for the different hypertensive subtypes at baseline compared to normotensives, stratified by sex.** Model 1: adjusted for age, sex, ethnicity and follow-up time. Model 2: model 1 + BMI, eGFR, smoking, diabetes mellitus, total cholesterol levels (all at baseline) and change in BMI. *ISH = isolated systolic hypertension. IDH = isolated diastolic hypertension. SDH = systolic diastolic hypertension. SA = South Asian Surinamese. A = African. BMI = body mass index. eGFR = estimated glomerular filtration rate. OR = odds ratio. CI = confidence interval. * Statistically significant (P < 0.05). ^a^ Diabetes mellitus is defined as elevated fasting glucose levels (≥7 mmol/l) and/or use of glucose lowering medication.*

|  | **A: Complete case analysis** | | **B: Imputation model** | | **C: Weighted regression analysis** | |
| --- | --- | --- | --- | --- | --- | --- |
|  | Men | Women | Men | Women | Men | Women |
| ISH | 4.8 (2.9 ; 7.8)* | 10.1 (4.1 ; 25.6)* | 4.8 (2,9 ; 7.9)* | 10.4 (4.2 ; 25.7)* | 4.8 (3.8 ; 6.0)* | 10.1 (7.1 ; 14.3)* |
| IDH | 6.0 (3.7 ; 9.7)* | 27.6 (14.7 ; 53.8) * | 5.7 (3.5 ; 9.5)* | 30.3 (15.9 ; 57.8)* | 5.9 (4.7 ; 7.3)* | 27.5 (21.4 ; 35.4)* |
| SDH | 33.7 (20.4 ; 58.4)* | 50.6 (24.8 ; 114.8)* | 31.8 (19.1 . 52,7)* | 45.7 (21.3 ; 98.2)* | 33.2 (26.4 ; 42.2)* | 50.0 (37.5 ; 67.7)* |

**Supplementary table 4: Sensitivity analysis on the effect of different methods to correct for loss to follow-up.** Values represent OR (95% CI) for risk of having sustained hypertension (SBP ≥ 140 mmHg, DBP ≥ 90 mmHg and/or use of antihypertensive medication) at follow-up for the different hypertensive subtypes at baseline compared to normotensives, stratified by sex. Model is adjusted for age, sex, ethnicity and follow-up time A: complete case analyses. B: imputation using multivariate imputation via chained equations (MICE). C: inverse probability weighted regression analysis. *ISH = isolated systolic hypertension. IDH = isolated diastolic hypertension. SDH = systolic diastolic hypertension. OR = odds ratio. * Statistically significant (P < 0.05).*

**
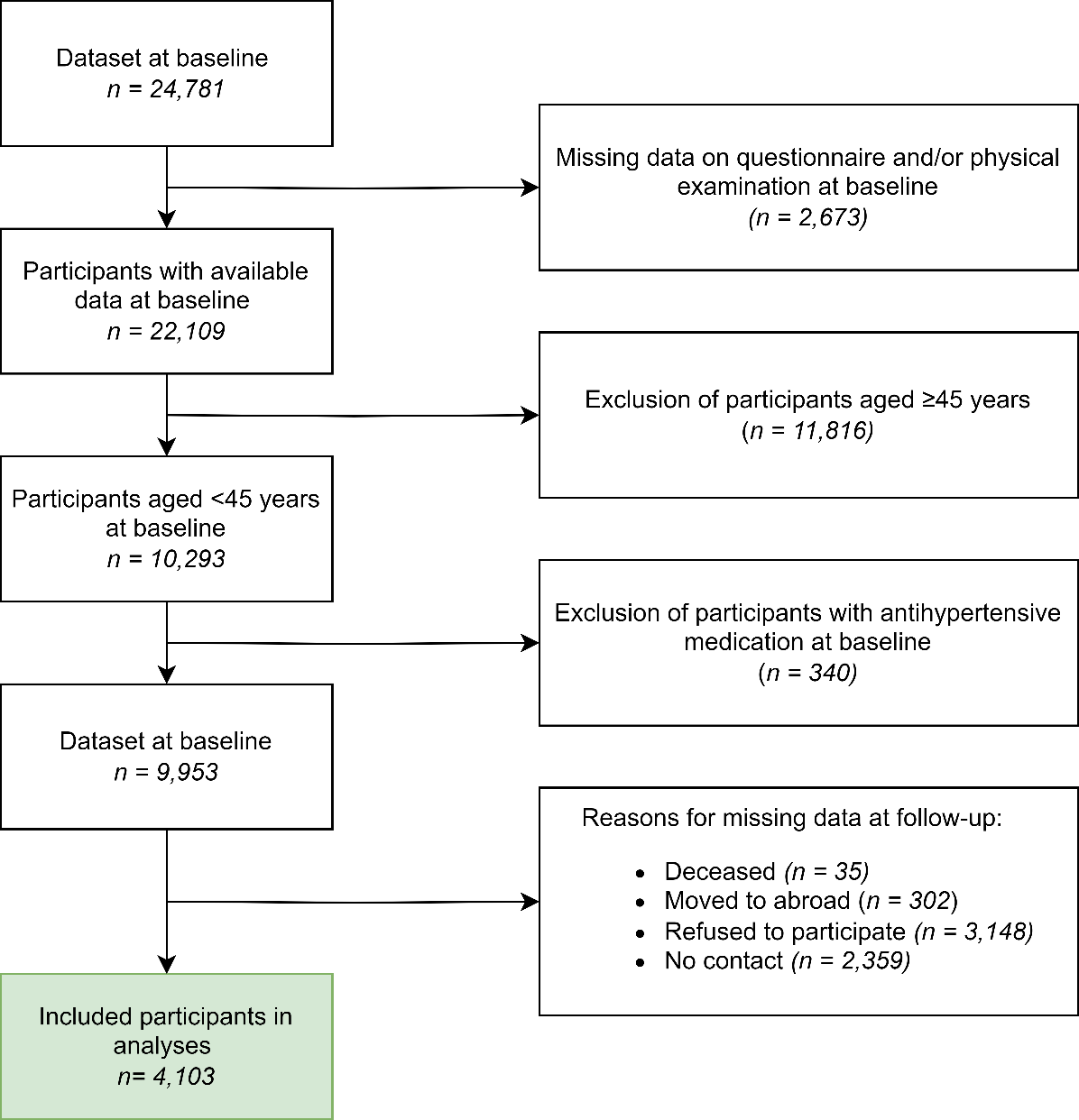
**

**Supplementary figure 1: Flowchart depicting selection of participants for the analyses.** *BP = blood pressure.*


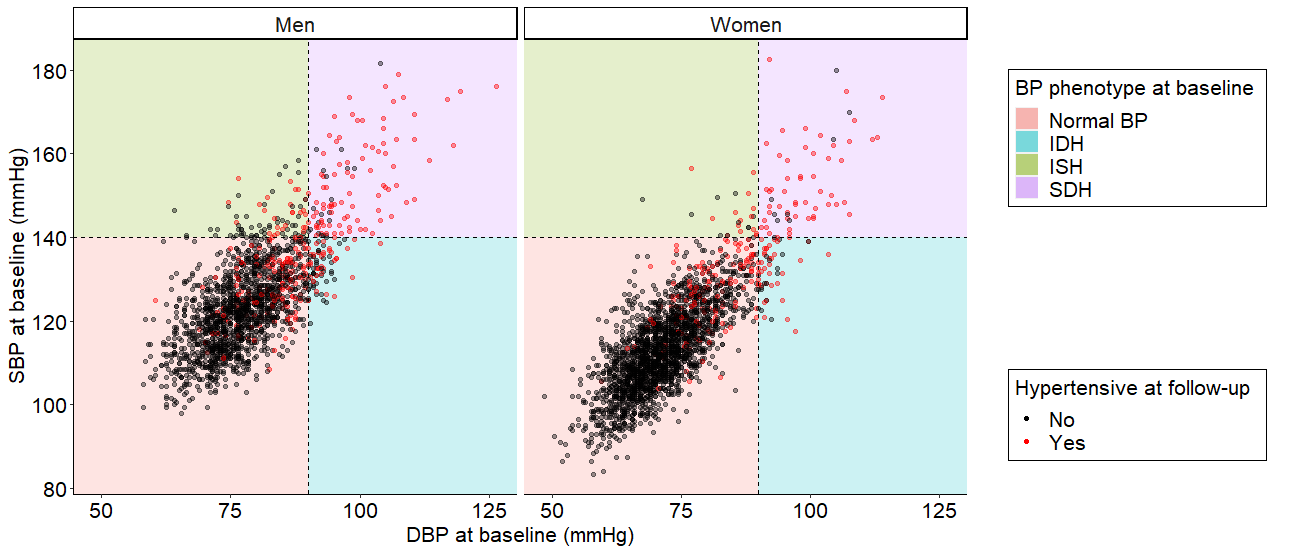


**Supplementary figure 2: Scatterplot of baseline BP levels categorised by BP phenotype.** Red dots represent the participants with hypertension (SBP ≥ 140 mmHg, DBP ≥ 90 mmHg and/or use of antihypertensive medication) at follow-up. *SBP = systolic blood pressure. DBP = diastolic blood pressure. ISH = isolated systolic hypertension. IDH = isolated diastolic hypertension. SDH = systolic diastolic hypertension.*
